# Supplementary material for: Lovastatin, an Up-Regulator of Low-Density Lipoprotein Receptor, Enhances Follicular Development in Mouse Ovaries
Source: Int J Mol Sci. 2023 Apr 14;24(8):7263. doi: 10.3390/ijms24087263 (PMC10139027; doi:10.3390/ijms24087263)
Supplement: Supplementary file 1 [file ijms-24-07263-s001.zip › ijms-2297705-supplementary.pdf]

International Journal of
Molecular Sciences

MDPI

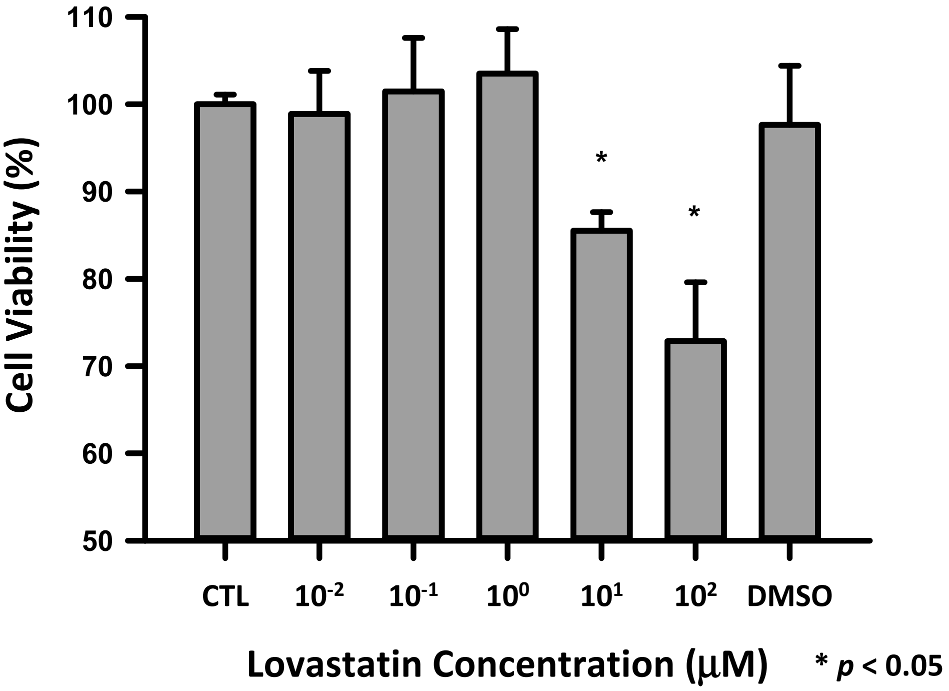

Figure S1. Cytotoxicity of lovastatin in the HEK293 cell line.

Table S1. RT-PCR and quantitative real-time PCR primers.

Primer	Gene		Sequence (5′-3′)
**LDLR**	Low density lipoprotein receptor	F	TGA TGG CCC CAA CAA GTT CA
		R	CTC GTT GGT CTT GCA CTC CT
**StAR**	Steroidogenic acute regulatory protein	F	TGT CTC CCA CTG CAT AGC TG
		R	CCG ATC CTT AGC ATC CCC TG
**AMH**	Anti-Mullerian hormone	F	CCA ATA CCA GGG GCC TCA TC
		R	GCT TGT GTT CCC CTC TCC TC
**GDF9**	Growth Differentiation Factor 9	F	AAA AGA CCA GGT GCC AGA GG
		R	GGT GGA CTG AAG AGA CTG CC
**BMP15**	Bone morphogenetic protein 15	F	CTC CTT GCT GAC GAC CCT AC
		R	GGT CAG CCG AAC GAT GGT AT
**Oct3/4**	Octamer-binding transcription factor 4	F	TGT TCA GCC AGA CCA CCA TC
		R	GCT TCC TCC ACC CAC TTC TC
**Nanog**	Nanog	F	GCA GAA GTA CCT CAG CCT CC
		R	ACC GCT TGC ACT TCA TCC TT
**Sox2**	SRY-Box Transcription Factor 2	F	AGG AGA GAA GTT TGG AGC CC
		R	TCT GGC GGA GAA TAG TTG GG
**β-actin**	beta-actin	F	CAT TGC TGA CAG GAT GCA GAA GG
		R	TGC TGG AAG GTG GAC AGT GAG G
